# Anion Inhibition Studies of the Beta-Carbonic Anhydrase from *Escherichia coli*

**DOI:** 10.3390/molecules25112564

**Published:** 2020-05-31

**Authors:** Sonia Del Prete, Viviana De Luca, Alessio Nocentini, Andrea Scaloni, Margaret D. Mastrolorenzo, Claudiu T. Supuran, Clemente Capasso

**Affiliations:** 1 Institute of Biosciences and Bioresources, CNR, Via Pietro Castellino 111, 80131 Napoli, Italy; sonia.delprete@ibbr.cnr.it (S.D.P.); vivianadeluca.81@gmail.com (V.D.L.); 2 Proteomics & Mass Spectrometry Laboratory, ISPAAM, CNR, Via Argine 1085, 80147 Naples, Italy, andrea.scaloni@ispaam.cnr.it; 3 Department of Neurofarba, Section of Pharmaceutical and Nutraceutical Sciences, University of Florence, Via U. Schiff 6, Sesto Fiorentino, 50019 Florence, Italy; alessio.nocentini@unifi.it (A.N.); maggie.mastrolorenzo@gmail.com (M.D.M.); 4 University of California, San Diego (UCSD), 3425 Lebon Drive, Unit 918, San Diego, CA 92122, USA

**Keywords:** carbonic anhydrase, anions, inhibitors, antibacterials, *Escherichia coli*, stopped-flow assay, protonography

## Abstract

The interconversion of CO_2_ and HCO_3_^−^ is catalyzed by a superfamily of metalloenzymes, known as carbonic anhydrases (CAs, EC 4.2.1.1), which maintain the equilibrium between dissolved inorganic CO_2_ and HCO_3_^−^. In the genome of *Escherichia coli*, a Gram-negative bacterium typically colonizing the lower intestine of warm-blooded organisms, the cyn operon gene includes the CynT gene, encoding for a β-CA, and CynS gene, encoding for the cyanase. CynT (β-CA) prevents the depletion of the cellular bicarbonate, which is further used in the reaction catalyzed by cyanase. A second β-CA (CynT2 or Can or yadF), as well as a γ and ι-CAs were also identified in the *E. coli* genome. CynT2 is essential for bacterial growth at atmospheric CO_2_ concentration. Here, we characterized the kinetic properties and the anion inhibition profiles of recombinant CynT2. The enzyme showed a good activity for the physiological CO_2_ hydratase reaction with the following parameters: k_cat_ = 5.3 × 10^5^ s^−1^ and k_cat_/K_M_ = of 4.1 × 10^7^ M^−1^ s^−1^. Sulfamide, sulfamate, phenylboronic acid, phenylarsonic acid, and diethyldithiocarbamate were the most effective CynT2 inhibitors (K_I_ = 2.5 to 84 µM). The anions allowed for a detailed understanding of the interaction of inhibitors with the amino acid residues surrounding the catalytic pocket of the enzyme and may be used as leads for the design of more efficient and specific inhibitors.

## 1. Introduction

*Escherichia coli* is a bacterium discovered in 1885 by the German bacteriologist Theodor Escherich who isolated it from the feces of a newborn [1]. This microorganism was initially named *Bacterium coli*, and lately, the term was modified to *Escherichia coli* to honor Escherich [1]. *Escherichia coli* is a harmless microbe, which typically colonizes the infant gastrointestinal tract within the first hours of life, establishing a mutual benefit with its host [2,3,4]. However, when the gastrointestinal mucosa is damaged by various factors affecting its integrity, the harmless microbe disseminates and provokes infection in the body, becoming a pathogen, which provokes a wide spectrum of diseases [2,5]. Although its discovery dates back to the previous century, only in 1935 *E. coli* was identified as the etiological agent responsible for the outbreak of diarrhea among infants [1]. The common genus *Escherichia coli* contains a broad variety of different forms: (i) pathogenic microorganisms, which can lead to death, or triggering severe disease outbreaks worldwide as well as serious infections, such as watery diarrhea, bloody diarrhea, urinary tract infection, meningitis, and sepsis [6,7,8]; (ii) opportunistic pathogens, which can cause disease if the host defenses are weakened [9]; and (iii) commensal microorganisms that innocuously colonize the healthy intestine of warm-blooded animals, including humans, with mutual benefits [10,11,12].

During their growth, bacteria need carbon dioxide (CO_2_) and bicarbonate (HCO_3_^−^), which are necessary for supporting the central metabolism [13,14]. The interconversion of inorganic CO_2_ and HCO_3_^−^ is naturally and correctly balanced to maintain the equilibrium between dissolved CO_2_ and HCO_3_^−^ [15,16,17,18]. The naturally occurring reaction of interconversion of CO_2_ and H_2_O into bicarbonate and protons (CO_2_ + H_2_O ⇌ HCO_3_^−^ + H^+^) cannot provide enough CO_2_/HCO_3_^−^ to the bacterium, being the reaction rate (k_cat_, catalytic constant) too low at physiological pH (k_cat hydration_ = 0.15 s^−1^ and k_cat dehydration_ = 50.0 s^−1^) [19,20]. Intriguing, the catalyzed CO_2_ hydration/dehydration reaction (CO_2_ + H_2_O ⇌ HCO_3_^−^ + H^+^) is the only known response of the bacterial metabolic pathway for rapidly obtaining and balancing the endogenous levels of CO_2_, H_2_CO_3_, HCO_3_^−^, and CO_3_^2−^ [13,14,21]. The catalyzed reaction has a k_cat_ ranging from 10^4^ to 10^6^ s^−1^ [22,23] and is carried out by a superfamily of ubiquitous metalloenzymes known as carbonic anhydrases (CAs, EC 4.2.1.1) [24,25,26,27,28]. The CA superfamily includes eight genetically distinct families (or classes), named with the Greek letters, α, β, γ, δ, ζ, η, θ, and ι [13,20,29]. The last three classes were only recently discovered [30,31]. Up to now, the exploration of the bacterial genome revealed only four of the eight CA-classes: α, β, γ, and ι [20,28,29,32,33,34,35,36], showing an intricate gene pattern distribution since the genome of some bacteria encodes for one, two, or even three different CA-families [13,20]. A fourth class, ι-CAs, recently discovered, was identified by our groups in the genome of *Burkholderia territorii*, which is a Gram-negative bacterium found in soil and water, which often shows resistance to common antibiotics [29,37]. In the genome of *Escherichia coli*, the cyn operon gene includes the CynT gene, encoding for a β-CA, and CynS gene, encoding for the cyanase, which catalyzes the reaction of cyanate with bicarbonate to give ammonia and carbon dioxide [38,39,40]. It has been hypothesized that the β-CA (CynT), catalyzing the CO_2_ hydration, prevents the depletion of the cellular bicarbonate, which thereafter will be used in the reaction catalyzed by the enzyme cyanase. Exploring the *E. coli* genome, a second β-CA (CynT2 or Can or yadF) was identified [41], whereas a γ-CA and a ι-CA (annotated as SgcJ/EcaC family oxidoreductase) were discovered by our groups (unpublished data from our laboratory and manuscript in preparation). CynT2 was characterized for its three-dimensional structure and for its essential role in allowing bacterial growth at atmospheric pCO_2_ [21,41]; no such information is available on the γ-and ι-CAs. In the latter context, it seems that the activity of *E. coli* CAs can promote bacterial growth and adaptation in the host. This observation is corroborated by the in vivo results demonstrating that CAs are crucial macromolecules for survival, pathogenicity, and virulence of several species of human pathogens, such as *Helicobacter pylori* [42,43,44], *Vibrio cholerae* [45], *Brucella suis* [46,47,48,49], *Salmonella enterica* [50], and *Pseudomonas aeruginosa* [51].

In this context, here, using a stopped-flow technique, we investigated the kinetic constants of the recombinant CynT2, a β-CA identified in the genome of *Escherichia coli*, for which the kinetic characterization has not yet been reported. Furthermore, since CynT2 is essential for bacterial growth at atmospheric CO_2_ concentration, its inhibition profile has been explored with a broad range of inorganic metal-complexing anions. These inhibitors are among the classical CA inhibitors (CAIs) and are very attractive because they are small molecules/ions, which can be efficiently transported in the body; in addition, they can exploit oxidation and ligand substitution reactions [52,53]. The aim of this work was to identify in vitro efficient inhibitors of CynT2, whose inactivation could impair the microbe diffusion in the host. Moreover, since *E. coli* is a microorganism that can be handled without risk in the laboratory, it might represent a bacterial study model to be used safely in vitro for cell-based tests, diversely from other human and animal pathogens, which require the use of particular levels of protection for reducing risks of contaminations. Moreover, we believe that the results of this study will be useful in further exploring novel approaches for the inhibition of bacterial CAs, which may lead to alternatives in the use of standard antibiotics for contrasting the growth and virulence of both human and animal pathogens.

## 2. Results and Discussion

### 2.1. Primary Structure Analysis

The genome of *Escherichia coli* was inspected with BLAST (Basic Local Alignment Search Tool) to identify the CA-classes encoded by the bacterial genome, using individually the amino acid sequences belonging to the eight CA-classes (α, β, γ, δ, ζ, η, θ, and ι) as query sequences. Table 1 summarizes the results obtained with the BLAST analysis. Three CA classes were identified in the *E. coli* genome: β, γ- and ι-CAs; furthermore, different isoforms were detected for each class of CAs.

**Figure 1:**
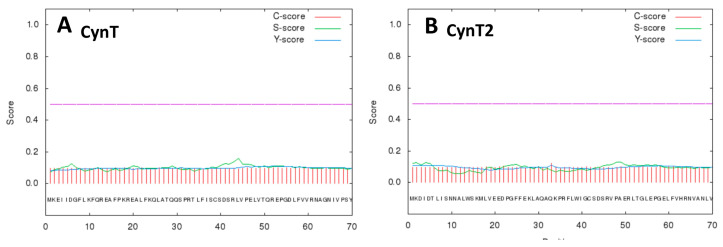
SignalP 4.1 graphical results obtained using the two β-CA polypeptide chains encoded by the *Escherichia coli* genome. Legend: Panel (**A**), CynT; Panel (**B**), CynT2; X-axis, the first 70 amino acid residues at the amino terminus of the polypeptide chain; Y-axis: C-score (red line), raw cleavage site score; S-score (green line); signal peptide score; Y-score (blue line), the combined cleavage site score. The violet line indicates the threshold value of 0.5.

Table 1 shows that no representative of the α-class was detected in the *E. coli* genome. In our previous works, it has been documented that the bacterial α-CAs identified in the Gram-negative bacteria are characterized by the presence of a short secretory or signal peptide at the N-terminal end of the polypeptide chain [13,19,29]. This short peptide allows the translocation of the neo-synthesized protein into the bacterial periplasmic space, which is a typical feature of the Gram-negative bacteria. Lately, this secretory signal was also identified in the amino terminus of some polypeptide chains of β-, γ-, and ι-CAs from Gram-negative bacteria [13,29]. These findings prompted us to investigate the N-terminal portion of the two *E. coli* β-CA sequences reported in Table 1 (CynT and CynT2, with the GenBank IDs WP_033547590.1 and EEW0221051.1, respectively) for the presence of a putative signal peptide. Figure 1 shows the results obtained using the bioinformatics tool “SignalP 4.1” (http://www.cbs.dtu.dk/services/SignalP/), which is a software optimized for the prediction of a signal peptide in Bacteria, Archaea, and Eukarya.

**Figure 2:**
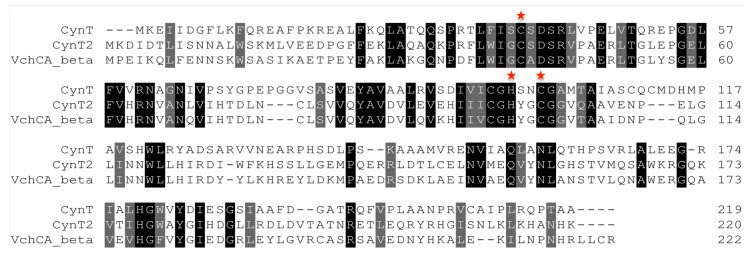
Multialignment of the two β-CAs (CynT and CynT2) from *E. coli and* the β-CA (VchCA_beta) from *Vibrio cholerae*. Legend: red stars indicate the metal ion coordinating residues. The alignment was formatted highlighting in black the identical residues and in gray the conservative substitutions. Multiple amino acid sequence alignment was performed with the program MUSCLE, version 3.7.

From Figure 1, it is readily apparent that all the scores represented in the SignalP output were very low, close to the value of 0.1. Thus, *E. coli* β-CAs seemed characterized by the absence of a secretory signal at the amino terminus of their sequence. The translocation of CAs in the space between the two bacterial membranes (outer and inner layers) of the Gram-negative bacteria guarantees the rapid conversion of the periplasmic CO_2_ to bicarbonate, avoiding its depletion. Furthermore, the bicarbonate transported in the cytoplasm will be used by the cytoplasmic CA-classes (β and γ) to produce the CO_2_/HCO_3_^−^ for the central bacterial metabolism [13,29]. From this consideration, it can be assumed that in *E. coli*, the lack of the α-CA is compensated by the presence of the ι-CA, whose polypeptide chain is typified by a secretory signal at the N-amino terminal [29]. This assumption is also corroborated by the existence of β- or γ-CAs with a signal peptide in Gram-negative bacteria, whose genome doesn’t encode for any α-CA. Figure 2 shows the multialignment of the two β-CAs amino acid sequences reported in Table 1 with a representative bacterial CA sequence belonging to the same family.

The isoform CynT showed an amino acid sequence identity of 28%, when compared with the isoform CynT2. The identity of CynT or CynT2 with the VchCA_beta from *Vibrio cholerae* was of 28% and 61%, respectively. This means that the two isoforms are characterized by multiple amino acid substitutions, even if the catalytic triad (two Cys and on His) is perfectly conserved (Figure 2). All the catalytically active CAs contain, independently of the genetic groups, a metal ion cofactor, which is necessary for enzyme catalysis [13,20,28,34]. The β-CAs use as catalytic metal a Zn^2+^ ion, which is coordinated by three amino acid residues; the fourth ligand is a water molecule/hydroxide ion acting as the nucleophile in the catalytic enzyme cycle, or an Asp residue in Type II β-CAs as CynT2 [41].

### 2.2. Three-Dimensional Structure Analysis

**Figure 3:**
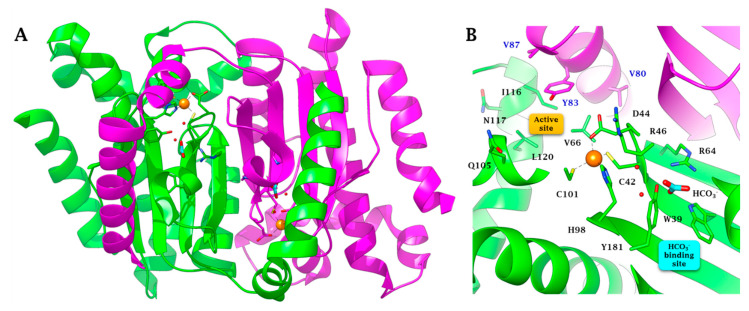
β-CA (CynT2, PDB 2ESF) from *E. coli*. (**A**) Ribbon representation of the dimer. (**B**) Zoomed view of the active site and bicarbonate binding pocket. The Zn(II) ion is represented as an orange sphere. Chain A and chain B are colored green and magenta, respectively. The HCO_3_^−^ ion is colored cyan. Amino acids composing the active site are labeled with one letter symbols (blue for chain A and black for chain B): A, Ala; C, Cys; D, Asp; H, His; I, Ile; L, Leu; N, Asn; Q, Gln; R, Arg; V, Val; W, Trp; Y, Tyr. The cyan box is the HCO_3_^−^ pocket, while the yellow box represents the catalytic pocket.

Figure 3 shows the X-ray crystal structure of the CynT2 from *E. coli*, which has been solved at 2.0 Å resolution; it reveals a dimeric arrangement of the protein, which is a tetramer, as two dimers interact with each other in the crystal packing [41]. The enzyme structure was determined as the closed conformation (type II β-CA) in which the zinc is tetrahedrally coordinated to Cys42, Asp44, His98, and Cys101 [41] (Figure 3A,B). The Asp44 side chain replacing the fourth ligand (water molecule) forms a non-canonical CA active site, which does not allow the CO_2_ hydration activity. This inactive form (closed active site) is present at pH values of less than 8.0 [41]. At pH values of ≥8.3, an incoming water molecule replaces the carboxylate moiety of the Asp residue, generating the nucleophile used in the typical catalytic cycle of the CAs [22,23]. Thus, the closed active site is converted to the open and active form (type I β-CA). As shown in Figure 3B, the closed structure is stabilized by HCO_3_^−^, which occurs in a non-catalytic binding pocket close to the zinc ion, as reported for other few β-CAs, such as those from and *Haemophilus influenzae* and *Vibrio cholerae* [54,55].

### 2.3. Production of Recombinat β-CA (CynT2)

**Figure 4:**
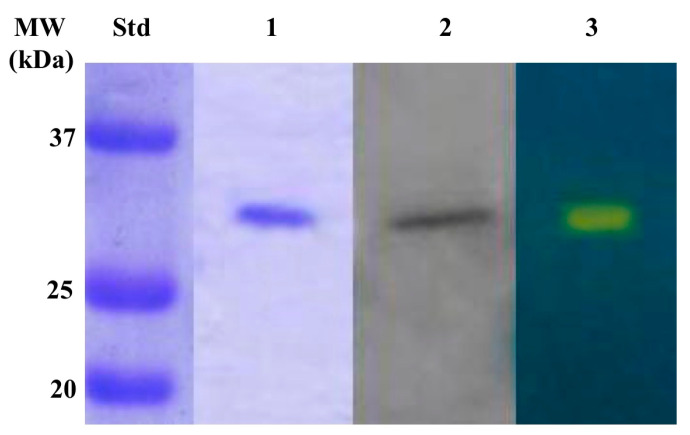
Combined results obtained with SDS-PAGE, Western blot and protonography, which were used to evaluate the recombinant CynT2 product purified by affinity column. The yellow band evidenced in the protonogram corresponds to the enzyme activity responsible for the drop of pH from 8.2 to the transition point of the dye in the control buffer. Lane STD, molecular markers (form bottom to the top: 20, 25, and 37 kDa); Lane 1, SDS-PAGE; Lane 2, Western Blot; Lane 3, Protonogram; Molecular markers (Lane STD).

We produced the bacterial CynT2 encoded in the *E. coli* genome, since a detailed investigation of its kinetic parameters is lacking to date, although, as mentioned above, the CynT2 three-dimensional structure has been solved [41]. Furthermore, this enzyme seems to be essential for bacterial growth at atmospheric pCO_2_ [21]. The purified enzyme, throughout the purification steps, was monitored following the Wilbur-Anderson Units (WAU) as described previously by Capasso’s group [56]. Figure 4 shows the purity of the recombinant CynT2 after the affinity column. Three biochemical techniques were used to verify the heterologous overexpression and purification of the bacterial enzyme, namely SDS-PAGE, Western Blot (WB), and protonography. SDS-PAGE and WB reported in Figure 4, (lane 1 and 2) indicated that the CynT2 fusion protein was purified to the homogeneity as a subunit with an apparent molecular weight of about 29.0 kDa. Moreover, the developed protonogram obtained by the protonography analysis evidenced that the recombinant enzyme was catalytically active (lane 3). It evidenced a yellow band due to the production of ions (H^+^) during the enzymatic CO_2_ hydration reaction at the molecular weight of 29.0 kDa, which corresponds to the mass of the recombinant CynT2 (Figure 4, lane 3).

### 2.4. Determination of the Kinetic Parameters Using the Stopped-Flow Technique

Using CO_2_ as a substrate, the recombinant CynT2 was subject to a stopped-flow analysis for the determination of the kinetic constants for the CO_2_ hydratase activity. As shown in Table 2, CynT2 showed a good biocatalyst activity for the physiological CO_2_ hydratase reaction to bicarbonate and protons, with k_cat_ of 5.3 × 10^5^ s^−1^ and catalytic efficiency (k_cat_/K_M_) of 4.1 × 10^7^ M^−1^ s^−1^. In fact, the CynT2 kinetic constants were similar to those obtained for other bacterial CAs, as well as for hCA I. CynT2 was also inhibited by the sulfonamide acetazolamide (K_I_ = 227 nM), which is a well-known pharmacological CA inhibitor (Table 2).

The results of Table 2 show that CynT2 was sensitive to 5-acetamido-1,3,4-thiadiazole-2-sulfonamide (acetazolamide, AZA) inhibition, similarly to the human isoform I (K_I_ = 250 nM), but it was 19 times less inhibited than the human isoform hCA II (K_I_ = 12 nM). Again, CynT2 with respect to VchCA_alpha from *V. cholerae* was 33 times less sensitive to AZA inhibition and 3.5 times less compared to the ι-CA from *B. territorii* (K_I_ = 65 nM). The comparison with the β-CA from *V. cholerae* (K_I_ = 451 nM) showed that the *E. coli* enzyme with a K_I_ of 227 nM is two times more susceptible to AZA inhibition. These results are of extreme importance in the field of the inhibition of bacterial CAs because they prove that, even if these enzyme catalyze the same reaction, they can show a different inhibition pattern, whose investigation can lead to the discovery of novel inhibitors, which may impair the microbial growth as well as their virulence [57].

### 2.5. Inhibition Profile of Inorganic Metal-Complexing Anions

Since CAs are crucial for bacterial growth as well as for their virulence, we decided to investigate the inhibition profile of CynT2 with a broad range of inorganic metal-complexing anions (Table 1).

Anions and small molecules, such as diethyldithiocarbamate, iminodisulfonate, sulfamide, sulfamate, phenylboronic and phenlylarsonic acids, can complex the metal ion (orange sphere of the Figure 3) of the enzyme catalytic pocket, hindering the hydration of the CO_2_ to bicarbonate and protons. They bind the Zn^2+^ ion of the enzyme either in a tetrahedral geometry or as trigonal-bipyramidal adducts of the metal ion [59]. Generally, these inhibitors show an inhibition constant (K_I_) in the millimolar range. Even if the anion inhibitors are usually less effective than sulfonamides (K_I_ is nM range), their investigation is essential for two fundamental aspects: (i) to design more efficient and selective inhibitors for the various CA-classes and their isoforms; (ii) potential for clinical applications for the treatment of diseases caused by pathogens, including bacteria. Here, we report the inhibition profile of CynT2 comparing these data with those obtained for the two human α-CAs, the isoforms hCA I and hCAII, and the bacterial enzyme (VchCA_beta), which have been previously investigated [58].

Table 3 lists the obtained results, which prompted us to elaborate the following observations:

1. A group of anions, such as iodide (I^−^), cyanide (CN^−^), azide (N_3_^−^), perchlorate (ClO_4_^−^) perosmate (OsO_5_^2^^−^), pyrophosphate (P_2_O_7_^2^^−^), divanadate (V_2_O_7_^2^^−^) perrhenate (ReO_4_^−^), hexafluorophosphate (PF_6_^−^), and trifluoromethanesulfonate (CF_3_SO_3_^−^) were very weak inhibitors of the *E.coli* β-CA, with a K_I_ > 10 mM (see Table 3). Within this group, VchCA_beta was inhibited with a K_I_ ranging from 5.7 to 9.0 mM (Table 3), except for OsO_5_^2^^−^, PF_6_^−^, and CF_3_SO_3_^−^, whose K_Is_ were even not measurable (Table 3). Moreover, N_3_^−^ and P_2_O_7_^2^^−^ showed a K_I_ > 10 mM for the CynT2, while ClO_4_^−^ with a K_I_ > 200 mM was an ineffective inhibitor of CynT2 as well as the *V. cholerae* enzyme. Interesting to note that some of these anions resulted to be quite effective inhibitors, when tested on the two human CAs. For example, cyanide and azide inhibited the human isoform h CA I with K_I_ of 0.5 and 12 µM, respectively (Table 3). Thus, low concentrations of N_3_^−^ and CN^−^ poison the two human enzymes as well as humans, whereas the bacterial CAs may better tolerate such toxic anions.
2. Another group of anion inhibitors, which weakly inhibited CynT2, showed a K_I_ in the range between 1.5 and 9.4 mM. This is the case of fluoride (F^−^), chloride (Cl^−^), bromide (Br^−^), thiocyanate (SCN^−^), nitrite (NO_2_^−^), nitrate (NO_3_^−^), bisulfite (HSO_3_^−^), sulfate (SO_4_^2^^−^), hydrogensulfide (HS^−^), selenate (SeO_4_^2^^−^), perruthenate (RuO_4_^−^), peroxydisulfate (S_2_O_8_^2^^−^), selenocyanate (SeCN^−^), iminodisulfonate (NH(SO_3_)_2_^2^^−^), and trithiocarbonate (CS_3_^2^^−^). The inhibitory behavior of these anions was very similar to that exerted on the enzyme from *V. cholerae* (VchCA_beta); exceptions were HSO_3_^−^, SO_4_^2^^−^, and NH(SO_3_)_2_^2^^−^, which were ineffective inhibitors for VchCA_beta with a K_I_ > 200 mM. At the same time, HS^−^ inhibited the *Vibrio* enzyme with a K_I_ > 20 mM. This last anion inhibited the human isoenzymes, hCA I and hCA II, with a K_I_ = 0.6 µM and K_I_ = 40 µM, respectively.
3. The submillimolar inhibitors of CynT2 showing a K_I_ in the range 0.25–0.89 mM were cyanate (CNO^−^), bicarbonate (HCO_3_^−^), carbonate (CO_3_^2−^), stannate (SnO_3_^2−^), tetraborate (B_4_O_7_^2−^), and fluorosulfonate (FSO_3_^−^). These anions were millimolar inhibitors for the VchCA_beta (Table 1). The differences in the values of the inhibition constants can be due to the amino acid residues surrounding the catalytic pocket, which influence the interaction of the anion with the enzyme, even if the three-dimensional structure of the β-CAs from *E. coli* and *V. cholerae* are very similar. Intriguing is the result obtained using bicarbonate and carbonate as inhibitors. These two anions are not effective inhibitors (K_I_ = 12–85 mM) for the two human isoforms (hCA I and hCA II), as well as for the β-CA from *V. cholerae* (K_I_ = 5.9–6.7 mM). These results may reflect an evolutionary adaptation of the human and *Vibrio* CAs due to their continuous exposition to the high concentration of these two anions. The human enzymes are adapted to the high concentration of carbonate and bicarbonate present in the plasma, while *V. cholerae* colonizes the upper part of the small intestine characterized by high concentrations of bicarbonate, which is also a potent inducer of the expression of the genes involved in the virulence of the pathogen [45].
4. The best inhibitors of CynT2 resulted to be the small molecules, such as sulfamide (NH_2_SO_2_NH_2_), sulfamate (NH_2_SO_3_H), phenylboronic acid (PhB(OH)_2_), phenylarsonic acid (PhAsO_3_H_2_) as well as diethyldithiocarbamate (EtNCS_2_^−^). As shown in Table 3, they showed K_I_ values from 2.5 to 84 µM. These inhibitors were also effective against VchCA_beta (K_I_ from 54 to 86 µM), except for EtNCS_2_^−^, which showed a K_I_ = 730 µM. Interesting to note, that dithiocarbamates were recently reported as a potent new class of CAIs targeting both the α- and β-classes of such enzymes [60,61,62].

From the mechanistic viewpoint, anions and small molecules as the ones investigated were shown to bind directly to the metal ion from the enzyme active site both for α- and β-CAs, as discussed extensively on the review by De Simone and Supuran [66] and by Whitesides’ group [67]. We estimate that anions investigated here show the same mechanism for the inhibition of CynT2 as the anions crystallized with various other α- and β-CAs.

## 3. Materials and Methods

### 3.1. Chemicals and Instruments

All the chemicals used in this study were of reagent grade and purchased from Sigma (Milano, Italy). The Affinity column (His-Trap FF) and the AKTA-Prime purification system were bought from GE Healthcare (Chicago, IL, USA). The SX20 Stopped-Flow was obtained by the AppliedPhotophysics. SDS-PAGE and Western-Blot apparatus were procured by BioRAD (Hercules, CA, USA).

### 3.2. Cloning, Expression, and Purification

The synthetic *Escherichia coli* gene encoding for the CynT2 was synthesized by the Invitrogen GeneArt (ThermoFisher Scientific, Waltham, MA, USA), a company specialized in gene synthesis, and cloned into the expression vector pET100D-Topo/CynT2. Briefly, the gene was designed to produce the recombinant CynT2 as fusion proteins with a tag containing nucleotides encoding for six histidines (His-Tag) at the amino terminus of neosynthesized recombinant protein. Competent *E. coli* BL21 (DE3) codon plus cells (Agilent) were transformed as described by Del Prete et al. [68]. Isopropyl β-D-1-thiogalactopyranoside (IPTG) at the concentration of 1 mM was added to the cellular culture to overexpress the recombinant CynT2. After growth, the cells were harvested and disrupted by sonication. Cellular extract was purified using a nickel affinity column (His-Trap FF), which allows the interaction between the matrix functionalized with Ni^2+^ ion and the His-Tag at the N-terminus of the protein. The HisTrap column (1 mL) was equilibrated with a 20-mL equilibration buffer (50 mM Tris, 20 mM imidazole and 150 mM sodium chloride, pH 7.5) at 1 mL/min. The supernatant from the cellular lysate was loaded onto the column at 1 mL/min, connected with AKTA Prime. The recombinant CynT2 was eluted from the column by fluxing a linear gradient of imidazole (0–300 mM) at a flow of 0.5 mL/min in a buffer composed of 50 mM Tris and 300 mM sodium chloride, pH 7.5. The recovered CynT2 was 90% pure. The protein quantification was carried out by Bradford method (BioRAD) [69].

### 3.3. Carbonic Anhydrase Assay for Monitoring the Recombinant Enzyme during the Purification Steps 

CA activity assay was performed as described by Capasso et al. [56]. Briefly, the assay was based on the monitoring of pH variation due to the catalyzed conversion of CO_2_ to bicarbonate. Bromothymol blue was used as the indicator of pH variation. The assay was performed at 0 °C and a CO_2_-satured solution was used as substrate. The enzyme activity was calculated by measuring the time required for Bromothymol blue to change from blue to yellow. This time is inversely related to the quantity of enzyme present in the sample and allows the calculation of the Wilbur-Anderson units as described previously [56].

### 3.4. SDS-PAGE

A 12% Sodium Dodecyl Sulfate-polyacrylamide gel electrophoresis (SDS-PAGE) prepared as described by Laemmli [70] was used, loading on the gel the recovered CynT2 from the affinity column. The gel was stained with Coomassie Brilliant Blue-R.

### 3.5. Western Blot

CynT2 was subjected to a 12% (*w*/*v*) SDS-PAGE, and then was transferred to a PVDF (polyvinylidene fluoride) membrane with transfer buffer (25 mM Tris, 192 mM glycine, 20% methanol) using Trans-Plot SD Cell (Bio-Rad, Hercules, CA, USA). His-Tag Western blot was carried out using the Pierce Fast Western Blot Kit (Thermo Scientific, Waltham, MA, USA). Blotted membrane was placed in the wash blot solution Fast Western 1 Wash Buffer to remove transfer buffer. Primary Antibody Working Dilution was added to the blot and incubated for 30 min at room temperature (RT) with shaking. Invitrogen anti-His antibody (1:10,000) was used. Afterwards, the blot was removed from the primary antibody solution and incubated for 10 min with the FastWestern Optimised HRP ReagentWorking Dilution. Subsequently, the membrane was washed two times in about 20 mL of FastWestern 1 Wash Buffer. Finally, the membrane was incubated with the detection reagent working solution and incubated for 1 min, at room temperature, and then developed with X-ray film.

### 3.6. Protonography

To perform the protonography, wells of 12% SDS-PAGE gel were loaded with samples mixed with loading buffer not containing 2-mercaptoethanol and not subjected to boiling, in order to avoid protein denaturation. The gel was run at 150 V until the dye front ran off the gel. Following the electrophoresis, the 12% SDS-PAGE gel was subject to protonography to detect the yellows bands due to the hydratase activity on the gel as described previously [71,72,73,74].

### 3.7. Kinetic Parameters and Inhibition Constants Determined by the Stopped-Flow Technique

The CO_2_ hydration activity performed by the BteCAι was monitored using an Applied Photophysics stopped-flow instrument [59]. Phenol red (at a concentration of 0.2 mM) was used as indicator, working at the absorbance maximum of 557 nm, with 20 mM TRIS (pH 8.3) as buffer, and 20 mM NaClO_4_ (for maintaining constant the ionic strength), following the initial rates of the CA-catalyzed CO_2_ hydration reaction for a period of 10–100 s. To determine the kinetic parameters by Lineweaver-Burk plots and the inhibition constants, a concentration of CO_2_ between 1.7 to 17 mM was used. At least six measurements of the original 5–10% reaction were used to assess the initial velocity for each inhibitor. The uncatalyzed rates were identically determined and detracted from the total observed rates. Stock inhibitor solutions (10–100 mM) were prepared in distilled-deionized water and dilutions up to 0.01 mM were done with the buffer test. Inhibitor and enzyme solutions were preincubated together for 15 min at room temperature prior to assay, in order to allow for the formation of the E–I complex or for the eventual active site mediated hydrolysis of the inhibitor. The inhibition constants were obtained by non-linear least-squares methods using PRISM 6 and the Cheng-Prusoff equation, as reported earlier [58,60,61], and represent the mean from at least three different determinations. All CA isoforms were recombinant ones obtained in-house.

## 4. Conclusions

The *E. coli* genome encodes only for three of the eight CA-classes reported in the literature: β-, γ-, and ι-CAs. In the present paper, we produced the recombinant isoform CynT2, a CA belonging to the β-class. The enzyme kinetic parameters and its anion inhibition profile were determined, using the stopped-flow technique. CynT2 resulted to be a good biocatalyst for the CO_2_ hydration reaction showing a k_cat_ = 5.3 × 10^5^ and a k_cat_/K_M_ = 4.1 × 10^7^. These values are similar to those obtained for other enzymes belonging to the β-CA class. Moreover, we also investigated the inhibition profile of CynT2 with a broad range of inorganic metal-complexing anions, a well-known group of CA inhibitors capable of blocking the enzyme activity. Small molecules such as sulfamide, sulfamate, phenylboronic acid, phenylarsonic acid, and diethyldithiocarbamate resulted to be the most effective CynT2 inhibitors (K_I_ = 2.5 to 84 µM). Despite these metal-complexing anions showed inhibition constants in the millimolar range, this anion investigation study is relevant, because it allowed a better understanding of the interaction of the CA inhibitors with the amino acid residues surrounding the enzyme catalytic pocket as well as the design of more efficient and specific inhibitors. Besides, it is important to keep in mind that the physiological role of CAs is to balance pH, CO_2_, and bicarbonate inside the bacterial cell, ensuring the right amounts of these molecules/ions to the bacterial metabolism. Thus, inhibition of such enzymes can impair bacterial growth as well as their virulence through a metabolic pathway, which is different from those used by the common pharmacological antibiotics. These could open a new solution to antibiotic resistance, which is occurring worldwide. Finally, *E. coli* is a microorganism that can easily be manipulated in the laboratory, avoiding the risk connected to the handling of pathogenic bacteria. Considering this aspect, *E. coli* could be considered as a model study organism for testing CA inhibitors in vitro, in cell-based assays, evaluating the effect of the inhibition on bacterial cell growth in a safe and facile way. To address the issues mentioned above, it is necessary to analyze in vitro the effect of CA inhibitors on all CA-classes encoded by the *E. coli* genome. That’s what we are doing.

## Figures and Tables

**Table 1:** CA-classes encoded by the *Escherichia coli* genome. This table reports only some representatives of the identified enzymes, such as two β, one γ, and ι-CAs.

CA-Class	Presence in Genome	Acronym	Number of Amino Acid Residues
α	Absent	-	-
β	Present	CynT	219
		CynT2	220
γ	Present	Ecoli_gamma	256
ι	Present	Ecoli_iota	150

- not applicable.

**Table 2:** CynT2 kinetic parameters for the CO_2_ hydration reaction. The CynT2 calculated kinetic constants were compared with those determined for the two human isoforms hCA I and II (α-class), the α-, β-, γ-CAs from *Vibrio cholerae* and ι-CAs from *Burkholderia territorii*. The reaction was performed at 25 °C, in 20 mM Tris buffer and 20 mM NaClO_4_, pH 8.3. Inhibition data with the clinically used acetazolamide are also provided.

Organisms	CA-Class	Acronym	k_cat_^1^ (s^−1^)	k_cat_/K_M_^1^ (M^−1^ × s^−1^)	K_I_ (Acetazolamide) ^1^ (nM)
*Homo sapiens*	α	hCA I **^2^**	2.0 × 10^5^	5.0 × 10^7^	250
	α	hCA II **^2^**	1.4 × 10^6^	1.5 × 10^8^	12
*Vibrio cholerae*	α	VchCA_alpha **^2^**	8.2 × 10^5^	7.0 × 10^7^	6.8
	β	VchCA_beta **^2^**	3.3 × 10^5^	4.1 × 10^7^	451
	γ	VchCA_gamma **^2^**	7.3 × 10^5^	6.4 × 10^7^	473
*Burkholderia territorii*	ι	BteCAι **^3^**	3.0 × 10^5^	9.7 × 10^7^	65
*Escherichia coli*	β	CynT2	5.3 × 10^5^	4.1 × 10^7^	227

^1^ Mean from 3 different assays by a stopped flow technique (errors were in the range of ±5–10% of the reported values); ^2^ From reference [58]; ^3^ From reference [29].

**Table 3:** Anions inhibition constants for the human α-CAs isoforms I and II, CynT2 (*E. coli* β-CA) and the β-CA (VchCA_beta) identified in the genome of *V. cholerae*. The analysis was carried out by a stopped-flow assay [63,64,65].

Anion	K_I_ (mM) ^1^			
	hCA I ^2^	hCA II ^2^	CynT2	VchCA_beta ^2^
F^−^	>300	>300	9.4	8.7
Cl^−^	6	200	6.7	8.1
Br^−^	4	63	3.8	7.4
I^−^	0.3	26	>10	9.0
CNO^−^	0.0007	0.03	0.58	7.1
SCN^−^	0.2	1.6	5.7	9.5
CN^−^	0.0005	0.02	>10	5.7
N_3_^−^	0.0012	1.51	>10	20.5
NO_2_^−^	8.4	63	4.9	9.1
NO_3_^−^	7	35	2.4	8.4
HCO_3_^−^	12	85	0.81	5.9
CO_3_^2−^	15	73	0.89	6.7
HSO_3_^−^	18	89	3.7	>200
SO_4_^2−^	63	>200	1.7	>200
HS^−^	0.0006	0.04	2.7	21.3
NH_2_SO_2_NH_2_	0.31	1.13	0.011	0.054
NH_2_SO_3_H	0.021	0.39	0.0025	0.086
PhAsO_3_H_2_	31.7	49	0.0061	0.079
PhB(OH)_2_	58.6	23	0.0028	0.085
ClO_4_^−^	>200	>200	>100	>200
SnO_3_^2−^	0.57	0.83	0.52	3.1
SeO_4_^2−^	118	112	3.1	3.4
TeO_4_^2−^	0.66	0.92	0.51	2.3
OsO_5_^2−^	0.92	0.95	>10	nt
P_2_O_7_^2−^	25.8	48	>10	15.1
V_2_O_7_^2−^	0.54	0.57	>10	7.9
B_4_O_7_^2−^	0.64	0.95	0.25	3.4
ReO_4_^−^	0.11	0.75	>10	6.3
RuO_4_^−^	0.101	0.69	9.5	8.4
S_2_O_8_^2−^	0.107	0.084	6.4	3.4
SeCN^−^	0.085	0.086	3.1	5.3
NH(SO_3_)_2_^2−^	0.31	0.76	1.5	>200
FSO_3_^−^	0.79	0.46	0.83	8.9
CS_3_^2−^	0.0087	0.0088	3.1	7.0
EtNCS_2_^−^	0.00079	0.0031	0.084	0.73
PF_6_^−^	-	-	>10	-
CF_3_SO_3_^−^	-	-	>10	-

^1^ Mean from 3 different assays by a stopped flow technique (errors were in the range of ±5–10% of the reported values); ^2^ From reference [58]; -: not detected.
